# Validation of the Hong Kong Chinese version of the Support Person’s Unmet Needs Survey—Short Form

**DOI:** 10.3390/ijerph16214103

**Published:** 2019-10-24

**Authors:** Doris Y. P. Leung, Yin-Ping Choy, Wai-Man Ling, Elaine Yim, Winnie K. W. So, Carmen W. H. Chan, Yim-Wah Mak

**Affiliations:** 1 School of Nursing, The Hong Kong Polytechnic University, Hong Kong, China; yw.mak@polyu.edu.hk; 2 Department of Oncology, Princess Margaret Hospital, Hong Kong, Chinayimpy@ha.org.hk (E.Y.); 3 Department of Clinical Oncology, Pamela Youde Nethersole Eastern Hospital, Hong Kong, China; lingwm1@ha.org.hk; 4 Nethersole School of Nursing, The Chinese University of Hong Kong, Hong Kong, China; winnieso@cuhk.edu.hk (W.K.W.S.);

**Keywords:** Chinese, cancer, caregiver, needs, SPUNS-SF

## Abstract

This study describes the psychometric properties of a Hong Kong Chinese version of the short form of the Support Person’s Unmet Needs Survey (SPUNS-SF) for caregivers of patients with cancer. A convenience sample of 280 patient-caregiver dyads was recruited between April and June 2018. A subsample of 70 caregivers completed the survey again, two weeks later. A confirmatory factor analysis (CFA) examined the instrument’s factorial structure, ordinal alpha coefficients evaluated the internal consistency, and intra-class correlation coefficients (ICCs) assessed the test-retest reliability. Convergence validity was evaluated by the correlations with sleep disturbance and caregiver burden in caregivers. The Hong Kong Chinese version of the SPUNS-SF (SPUNS-SFHKC) had a high completion rate of 96.8% (271/280) among caregivers. The original five-factor model provided an acceptable fit with the data in the CFA. The ordinal alpha coefficients were 0.866–0.945, and the two-week test-retest reliabilities were 0.524–0.678. The correlations of the five domains of the SPUNS-SFHKC with caregiver burden were 0.257–0.446, and for sleep disturbance were 0.075–0.464. The SPUNS-SFHKC has a suitable factor structure and psychometric properties for use in assessing unmet supportive needs among Chinese caregivers of patients with cancer. The applicability of the instrument for long-term use still needs to be studied.

## 1. Introduction

The incidence of cancer in Hong Kong has been increasing. In 2016, the number of new cancer cases reached 31,468, with 50% having been diagnosed at age ≥ 65 [1]. With advancements in treatments, cancer patients are living longer, but their well-being might be poor. The pressure is being put on already tight medical resources. Indeed, cancer was the second major cause of inpatient attendances in 2014, accounting for 12.2% of the total number of attendances in all hospitals [2]. With the aging of the population and long life expectancies, the cancer burden in Hong Kong is growing tremendously [3].

Cancer is among the most common conditions requiring supportive care from informal caregivers. The increasing number of long-lived cancer survivors and the high cost of cancer treatments have shifted the responsibility for cancer care from the hospital to the community, and are leading to an unprecedented dependence on caregivers to provide support for high-quality care [4]. The transition to the caregiving role, however, is life-changing, with many caregivers often taking up important and demanding roles and responsibilities with little or even no formal training. This makes them vulnerable as a group to chronic diseases. Many cancer caregivers have reported that caregiving had a negative impact on their own well-being. It has been reported that cancer caregiving can lead to physical impairments [5], and is associated with psychological impairments, sleep disturbances, financial issues, and poor quality of life (QoL) [6]. Furthermore, caregivers of patients with cancer experienced a similar burden and psychological distress to that of caregivers of patients with dementia [7]. In addition, caregivers had poorer QoL, experienced greater fear of the recurrence of cancer, and had less family support than the patients themselves [8]. A recent local study on 231 caregivers of cancer patients also found that 66% had poor physical well-being, and 54% had poor mental well-being [9]. With the increasing number of cancer survivors and a global demographic shift, there will be an ever-increasing cohort of caregivers who will need assistance to sustain their role.

To engage effectively in the caregiving process, caregivers have to assume a complex and multifaceted role, which may include providing a wide range of types of support to the patient [10]. While support from a caregiver reduces the demands on the healthcare system, all over the world, the caregiver’s own physical, emotional, psychological, and financial needs are largely neglected [11,12,13,14]. These unmet needs change over time, from the need for information about the patient’s cancer diagnosis in the earlier stages to well-being and relationship needs in the later stages [13,15]. Although effective interventions for cancer caregivers have been developed, they are rarely implemented in practice, which might be due to a lack of awareness of the needs of caregivers in clinical practice or to a lack of trained professionals to implement the interventions [6]. In Hong Kong, cancer patients are treated and followed up at outpatient clinics. They are expected to take care of themselves with the help of family members or friends throughout the treatment and rehabilitation stages. Although a wide range of types of support are available, caregivers themselves might not be aware that they have such needs or that there are such services to meet their needs, leading to a service gap and to the underutilization of valuable resources [9]. For example, Chinese cancer patients might take over-the-counter Chinese herbal products or cook some kinds of fortifying soup to maintain their health [16], but the related cost is not covered in the current healthcare system. A local qualitative study on the parents of children with cancer also reported they had unmet supportive care needs in four domains, namely, informational, psychosocial, spiritual, and financial [17]. Hence, it is important to develop tools for assessing the unmet needs of caregivers as a mechanism for identifying gaps in services and priorities in the development of services. As yet, such a tool is lacking for the caregivers of cancer patients in the Hong Kong Chinese population.

A recent review [18] identified a few psychometrically robust measures designed specifically to assess the unmet supportive care needs of caregivers of patients with cancer. It concluded that the longer 78-item Support Person’s Unmet Needs Survey (SPUNS) [19] appears to be the most appropriate for research purposes, but is too long for clinical practice. Later, a shorter version of SPUNS (SPUNS-SF), containing 26 items, was produced and shown to have good reliability, validity, and responsiveness [11]. The SPUNS-SF was further translated and validated in several places, including Mexico and mainland China [20,21]. The preliminary evidence supports the use of the SPUNS-SF to measure the unmet needs of the caregivers of cancer patients [11,20,21], but the scale has not been psychometrically tested with Hong Kong Chinese samples.

Han and colleagues [21] developed a Chinese version of the SPUNS-SF for use in mainland China, but it is unclear whether this scale would be equally applicable to Hong Kong Chinese. Hong Kong has been a British colony for about 150 years. Hong Kong’s citizens have been living within a Western administrative-legal framework, and Hong Kong has become a highly modernized commercial center in Asia. With the process of Westernization, the predominant family structure has changed from the traditional extended family to a nuclear family of about 3.1 persons, which may have an impact on caregiving patterns as compared to mainland China [22]. In addition, Hong Kong and mainland China have very different languages and cultures [23]. The characters used in mainland China are not always the same as those used in Hong Kong [24], which means that people in Hong Kong may not understand some words used in mainland Chinese. Thus, the purpose of this study is to validate a Hong Kong Chinese version of the 26-item Support Person’s Unmet Needs Survey—Short Form (SPUNS-SF) to provide a practical tool for assessing the unmet support needs of the caregivers of cancer patients in Hong Kong. The aim of this study is to evaluate the psychometric properties of a Hong Kong Chinese version of SPUNS-SF for use among caregivers of cancer patients. 

## 2. Materials and Methods

### 2.1. Study Design, Sample, and Participants

This was a methodological study consisting of a cross-sectional survey and follow-up survey with a subsample, which was conducted in Hong Kong between April and June 2018. A convenience sample of patient-caregiver dyads was recruited in the oncology outpatient clinic of two hospitals. Eligible patients were those who satisfied the following criteria: (1) aged 18 or above; (2) diagnosed with cancer; (3) at any stage of treatment for cancer; (4) medically stable; and (5) able to communicate in Chinese. Patients were excluded if they were mentally incompetent at the time of recruitment and could not nominate a family member or friend to take part in the study. Patients were asked to nominate a caregiver to participate in the study. The criteria for the eligibility of the caregiver to join this study included: (1) age 18 or above; (2) have been taking care of the patient for the past three months; (3) an unpaid caregiver as suggested by the patient; (4) with no diagnosis of cancer; and (5) who is Chinese. The study was approved by the Research Ethics Committee, Kowloon West Cluster (KW/EX-17-096(113-05)), the Hong Kong East Cluster Research Ethics Committee (HKECREC-2017-033); and the Joint Chinese University of Hong Kong-New Territories East Cluster Clinical Research Ethics Committee (CREC.2017.339). The written informed consent of the eligible patient-caregiver dyads was obtained before the questionnaire was administered. 

### 2.2. Sample Size Determination

A sample of 280 patient-caregiver dyads was recruited for the study. Such a sample size (> 200) was sufficient for accurate inferences in confirmatory factor analysis [25] and allowed for a small percentage of incomplete or problematic questionnaires [26]. For the assessment of the two-week test-retest reliability of the translated SPUNS-SF, assuming that the true reliability is 0.8 and the observed reliability is 0.7, a total of 66 subjects would be needed to achieve a power of 0.95. Again, allowing for a small percentage of incomplete and problematic questionnaires, we recruited a test-retest subsample of 70 caregivers at two weeks. 

### 2.3. Measures

Three instruments were administered to the caregivers to measure their unmet supportive care needs in caregiving, sleep disturbance, and caregiving burden, respectively. 

The SPUNS-SF consists of 26 items measuring the unmet needs of caregivers in taking care of cancer patients. These items are categorized under five domains: Information and relationships (6 items), Personal and emotional needs (seven items), Work and finances (five items), Healthcare access and continuity (five items), and Worries about the future (three items). The participants were asked to respond to the items based on a five-point Likert scale: 0 = “no unmet needs” to 4 = “needs are very much unmet.” Subscale scores were calculated by averaging the corresponding items in the respective subscale. The subscale scores ranged from 0 to 4, with higher scores indicating higher levels of unmet supportive care needs. The SUNPS-SF was shown to have good psychometric properties in a sample of 1,183 caregivers of cancer survivors, with the Cronbach’s alpha values of the five domains ranging from 0.87 to 0.94, and the factor structure similar to the original version of the scale [11].

The Pittsburgh Sleep Quality Index (PSQI) is a 19-item self-reported instrument measuring sleep disturbances during the past month. A global score for sleep disturbance is calculated from seven components (range: 0–21), with a higher score indicating poorer sleep quality. The Chinese version of PSQI was shown to have acceptable internal consistency and construct validity in breast cancer patients [27]. Previous studies had reported that cancer caregiving was associated with poor sleep quality [6].

The Chinese version of the 13-item Caregiver Strain Index (CSI) [28] was used to assess the global burden of family caregivers. The CSI has been widely used in studies on the burden of caregivers of patients with chronic illnesses. Items are rated based on a yes (1) or no (0) response, with the total score ranging from 0–13, and higher scores indicating greater levels of burden. The CSI has been shown to have good psychometric properties in a sample of caregivers in the community [28].

### 2.4. Procedure

Permission to translate the SPUNS-SF into Chinese was obtained from the developers of the scale. The scale was translated into Chinese using the World Health Organization’s four-step guideline on instrument translation and adaptation [29]. The SPUNS-SF was first forward translated by a nurse in palliative care, and the draft translation was assessed by an expert panel. Then, another nurse who was blinded to the original version of SPUNS-SF did a backward translation. The back-translated SPUNS-SF was then assessed by the expert panel, with discrepancies identified and resolved by discussion or by reviewing the translation procedure until a satisfactory conceptual and semantic equivalent was found. The translated instrument was then pre-tested with interviews with 10 primary caregivers to achieve idiomatic and experiential equivalence. The final version of the Hong Kong Chinese version of SPUNS-SF (SPUNS-SFHKC) (Table S1) has good content validity, with a content validity index score of ≥0.95 at the item level.

Trained research assistants (RAs) approached and screened patients and their caregivers in the outpatient clinics for eligibility. The RAs administered the questionnaires to both the patients and caregivers, but some of the caregivers completed the questionnaires by themselves. At two weeks, a convenience subsample of 70 caregivers was selected, and the RA administered the SPUNS-SFHKC again via telephone. In the study, it took less than 5 minutes for the caregivers to complete the SPUNS-SFHKC.

### 2.5. Data Analysis

A confirmatory factor analysis (CFA) was conducted to assess the original factor structure of the SPUNS-SFHKC. A five-factor model was fitted to the data of the scale using the EQS package [30]. Satorra and Bentler’s robust maximum likelihood estimation procedure is recommended for use in the ordinal CFA when the number of response categories for each item in the scale is equal to or greater than five [31]. The model was first fitted without allowing for any residual correlations among items. Then, Lagrange Multiplier (LM) tests were used to identify item pairs that would improve model fit if they were allowed to correlate and if the modification was theoretically plausible. It was determined that the model was a good fit with the data, according to the results of several fit indices including (a) a standardized root mean squared residuals (SRMR) score of ≤0.08, (b) a robust Comparative Fit Index (R-CFI) score of ≥0.90, and (c) a robust root mean square error of an approximation (R-RMSEA) score of ≤0.08 [32].

Ordinal alpha coefficients, recommended for ordinal variables, were calculated to evaluate the internal consistency of each domain in the scale [33]. The test-retest reliability of the scale scores at two weeks was assessed using an intra-class correlation coefficient (ICC). For each domain, if more than 30% of the caregivers obtained the highest (4) or lowest (0) score, then ceiling or floor effects were inferred, respectively [26]. 

The convergent validity of the scale was examined by correlations with PSQI and CSI scores. The strength of the correlations was classified as weak (< 0.3), moderate (0.3–0.7), or strong (> 0.7) [34]. The previous study in China reported low-to-moderate correlations between SPUNS-SF domains and caregiving burden (range: 0.24–0.63) [21]; hence, we hypothesized that all five domains of SPUNS-SFHKC would correlate positively with CSI to a moderate extent. On the other hand, we hypothesized that there would be low correlations between SPUNS-SFHKC and PSQI since studies have reported that caregivers tend to underrate the extent of their sleep disturbance [35], and some might regard their sleep disturbance as insignificant when compared with the cancer patient’s illness [36]. All statistical analyses, with the exception of CFA and ordinal alpha coefficients by EQS, were performed using SPSS24.0 with the significance level set at 0.05.

## 3. Results

### 3.1. Participant Characteristics

Overall, 72.4% (330/456) of eligible patients and 95.5% (234/245) of eligible caregivers provided their full informed consent and participated in the study. At two weeks, a subsample of 70 caregivers completed the SPUNS-SFHKC again. Table 1 shows the demographic characteristics of 280 caregivers. Their mean age was 50.0 years (SD = 14.7), 33.6% were male, 87.1% had secondary education or above, and 36.1% and 37.5% were the child and spouse of the patient, respectively. Only 7.2% reported having poor health status, 62.9% lived with the patient, and about half had been caring for the patient for less than two years. Regarding the average daily time spent in caring for the patient, 30.7% reported that it was more than seven hours. The re-test sample well resembles the test sample in all aspects except that more children were the caregivers in the re-test sample.

Table 2 shows the demographic and clinical characteristics of the patients in the test and re-test samples. Among the 280 patients, the mean age was 60.9 years (SD = 13.2), 33.6% were male, 68.2% had secondary education or above, and 30.7% perceived their financial status as poor. Regarding their clinical characteristics, the top three types of cancer were breast cancer (28.2%), colorectal cancer (18.6%), and lung cancer (16.1%). About half of the patients had had cancer for less than two years, and 87.9% had undergone treatment. Again, the re-test sample resembled the test sample in all aspects, except that those in the re-test sample had a lower level of education. 

### 3.2. Missing Data of SPUNS-SFHKC

The rate of missing data for the SPUNS-SFHKC was extremely low, with one caregiver not having responded to 11 items (items 15 to 25), two caregivers not responding to three items (items 24 to 26), and six caregivers not responding to one of the 26 items. Thus, the data from 271 (96.8%) patient-caregiver dyads at baseline and from all 70 caregivers at two weeks were used in the analysis. 

### 3.3. Confirmatory Factor Analysis

A five-factor model was fit for the SPUNS-SFHKC items, and the fit indices suggested an adequate fit to the data (SRMR = 0.078, R-CFI = 0.884 and R-RMSEA = 0.081). LM tests indicated an improvement in model fit if three pairs of item errors were allowed to correlate; between item 20 (“not sleep well”) and item 21 (“dealing with feeling stressed”) in the Personal and emotional domain, and between item 10 (“getting my boss to be more supportive and understanding”) and item 13 (“dealing with the way co-workers feel about my situation”) and between items 12 (“paying non-medical costs (such as travel, special food)”, and item 14 (“finding and getting financial help”) in the work and finances domain. The model was refitted with specifications for the three pairs of correlated item errors, thereby improving the model fit (SRMR = 0.074, R-CFI = 0.934 and R-RMSEA = 0.061). Standardized factor loadings for the items were high, ranging from 0.635 to 0.974, and the three error correlations were strong, at 0.577, 0.579, and 0.721 for item pairs 20 and 21, 10 and 13, and 12 and 14, respectively.

### 3.4. Internal Consistency and Test-Retest Reliability 

The resulting SPUNS-SFHKC demonstrated good internal consistency and acceptable test-retest reliability, with ordinal alpha coefficients of > 0.86 and ICC values of > 0.52 over a two-week interval for the subsample of 68 caregivers (Table 3). The proportion of caregivers who obtained the lowest (0) score ranged from 25.1% (Work and finances) to 7.7% (Personal and emotional needs), whereas the proportion of those who obtained the highest score (4) ranged from 0.4% (Personal and emotional needs) to 6.3% (Worries about the future), showing the absence of floor and ceiling effects. 

### 3.5. Convergent Validity

Table 4 presents the correlations of the five domains of SPUNS-SFHKC with the caregivers’ PSQI and CSI scores. Five out of the 10 hypothesized relationships of the SPUNS-SFHKC domains with CSI and PSQI were confirmed by showing moderate correlations. Regarding the hypothesized positive relationship with CSI, moderate correlations were found in all of the five domains of the SPUNS-SFHKC, with the exception of the Information domain, which was low. Regarding the hypothesized positive relationship with PSQI, the Personal and emotional domain showed a moderate relationship, while low correlations were found in the other four domains. Only one of these low correlations was found to be non-significant. 

## 4. Discussion

This was the first study to use a confirmatory approach to examine the psychometric properties of the SPUNS-SFHKC using a sample of Chinese caregivers of cancer patients recruited from hospitals. This study provides robust evidence of the validity and reliability of the Hong Kong Chinese version of the SPUNS-SF. The completion rate for the SPUNS-SFHKC was very high, and the caregivers completed the instrument within a few minutes with no difficulty, suggesting that it is easy to administer and puts a little burden on caregivers. Hence, the instrument could be used to measure the unmet needs of caregivers of cancer patients, even in a busy clinical setting. 

The results of the confirmatory factor analysis supported the five-factor structure of the Hong Kong Chinese version of the SPUNS-SF in this study. This finding resembled the original factor structure of the scale as proposed by Campbell and colleagues [19], but was contrary to that of the two validation studies on the mainland Chinese and Mexican versions of the SPUNS-SF [20,21], in which the factor structures reported in both studies derived from the original Canadian sample. The discrepancy may be due to the use of a different analytic approach (confirmatory versus exploratory), but also possibly because of the deletion of the work-related items before the analysis was conducted in these two previous studies. Although a five-factor structure was reported in the mainland Chinese sample, three items (10, 11, and 13) in the Work and finances domain and two items in the personal and emotional needs domain (24 and 26) were removed before the exploratory factor analysis was conducted, because a high proportion of respondents reported that they had no unmet needs on these five items. Similarly, for the Mexican study, three items (10, 11, and 13) in the work and finances domain were removed before the analysis, resulting in a six-factor structure. This discrepancy may be due to the deletion of the work-related items (items 10, 11, and 13) in the work and finances domain of the scale in these two previous studies, leaving only two finance-related items in the domain to be included in the exploratory factor analyses. There may be concrete explanations for the high proportion of respondents in these two previous studies who indicated that they had no unmet needs in these three work-related items. In the case of Mexico, there may be some health service schemes that allow caregivers to focus on caregiving by providing full monetary support to patients, whereas employees in China can request a short period of leave with basic pay, and caregivers and patients are usually well supported and cared for by family members because several generations of a family usually reside together in one house.

It is interesting to note the two pairs of error covariance in the Work and finances domain and one pair in the Personal and emotional needs domain in the 5-factor model in the current study. Compared to the two previous studies [20,21], the unmet needs in the work-related items are expected to be higher in Hong Kong because of the family structure and healthcare system. The size of the average household has shrunk to about 3 persons; hence, the nuclear family has become the predominant type of family [22], which might make it difficult to provide supportive care to family members. Moreover, as the healthcare system in Hong Kong only covers basic medications, many cancer patients and their families have to pay for cancer-related medications, although patients can always have access to the healthcare system when they are sick. A previous study also reported that Chinese cancer patients undergoing chemotherapy or radiotherapy might practice self-care activities underpinned by the philosophy of traditional Chinese medicine—that is, they might take over-the-counter Chinese herbal products or cook some kinds of fortifying soup to maintain their health [16]. Thus, keeping a job may be very important for caregivers to pay for the patient’s treatment costs. Although we did not collect information on the employment status of the caregivers, the proportion of respondents in the current sample who reported having no unmet needs in these three work-related items was comparatively low, ranging from 49.4% to 66.3%. This particular situation in Hong Kong might provide a partial explanation for the two pairs of error covariance in the Work and finances domain in our sample. The presence of the correlation between item 20 and item 21, on the other hand, might be due to the co-existence of two depression-related symptoms. Nevertheless, correlated errors in the items would be an area of interest for further study.

In our study, the ordinal alpha coefficients of the SPUNS-SFHKC exceeded 0.8, and hence, were considered to be good, showing the internal consistency of the instrument. In addition, the ICCs of the five domains of the scale that were observed during a two-week period were in the middle of the range of 0.4–0.75, indicating fair to good stability [37]. Nevertheless, this finding extends the previous study showing the good psychometric properties of the Hong Kong Chinese version of the SPUNS-SF. 

SPUNS-SFHKC has convergent validity, as shown by the moderate correlations of personal and emotional needs with both the caregiving burden and sleep disturbance, as well as between future concerns about needs and the caregiving burden. Compared with a previous validation study conducted in China [21], the magnitude of the correlations between the domains of the SPUNS-SFHKC and the caregiving burden are similar, except that the correlation with personal and emotional needs was higher in their study (r = 0.63). As expected, low correlations in the domains in the SPUNS-SFHKC with sleep disturbance were observed in the current study, with the exception of a moderate correlation with the Personal and emotional domain. This moderate correlation could be explained by findings in the literature, which report that anxiety, depressed mood, and stress could be related to sleep disruptions [38]. Alternatively, it might be due to some overlap in the contents of the items, as item 20 of the SPUNS-SF is also on the issue of sleep. These findings are consistent with previous results [20], and provide evidence of the construct validity of the SPUNS-SF. 

There are several limitations in the current study that are worth noting. First, the five-factor solution for the SPUNS-SFHKC was replicated in a single sample, and a cross-validation study using new samples and a confirmatory approach would be necessary to confirm its factor structure further. Second, further studies should be conducted to continue to evaluate the psychometric properties of the SPUNS-SFHKC, particularly with regard to the properties that were not assessed in the present study, such as predictive validity over time. Third, the use of a convenience sample with a relatively small sample size in the current study limits the generalizability of the study findings.

## 5. Conclusions

The results of the study with a Hong Kong Chinese sample of caregivers of people with cancer provided evidence of the good psychometric properties of the SPUNS-SFHKC. The scale appears to be a practical instrument for measuring the unmet needs of Chinese caregivers of patients with cancer. Healthcare professionals in cancer care may now have a practical and usable instrument. More studies examining the psychometric properties of the scale are warranted to establish the applicability of the instrument for long-term use.

## Figures and Tables

**Table 1:** Characteristics of primary caregivers.

Characteristics	Test Sample (*N* = 280)	Retest Sample (*N* = 70)
Age, mean ± SD	50.0 ± 14.6	47.7 ± 13.5
Male, *n* (%)	94 (33.6%)	17 (24.3%)
Married/Cohabiting, *n* (%)	209 (74.6%)	50 (71.4%)
Educational level, *n* (%)		
No formal education	4 (1.4%)	0 (0%)
Primary education	32 (11.4%)	3 (4.3%)
Secondary education or above	244 (87.1%)	67 (95.7%)
Perceived poor financial status, *n* (%)	59 (25.4%)	21 (30.0%)
Relationship with the patient, *n* (%)		
Child	101 (36.1%)	34 (48.6%)
Spouse	105 (37.5%)	17 (24.3%)
Relatives	50 (17.9%)	8 (11.4%)
Friend	11 (3.9%)	6 (8.6%)
Others	13 (4.6%)	5 (7.1%)
Perceived poor health status, *n* (%)	20 (7.2%)	3 (4.3%)
Living with the patient, *n* (%)	176 (62.9%)	41 (58.6%)
Have a domestic helper to help in caring for the patient, *n* (%)	35 (12.5%)	9 (12.9%)
Years of caring for the patient, *n* (%)		
< 1 Year	84 (30.0%)	24 (34.3%)
1–< 2 Years	71 (25.4%)	16 (22.9%)
2–3 Years	40 (14.3%)	10 (14.3%)
≥ 3 Years	79 (28.2%)	19 (27.1%)
Missing	6 (2.1%)	1 (1.4%)
Average time spent in caring for the patient per day, *n* (%)		
< 1 Hour	26 (9.3%)	8 (11.4%)
1–< 3 Hours	63 (22.5%)	20 (28.6%)
3–< 5 Hours	46 (16.4%)	16 (22.9%)
5–< 7 Hours	36 (12.9%)	5 (7.1%)
≥ 7 Hours	86 (30.7%)	17 (24.3%)
Missing	23 (8.2%)	4 (5.7%)
Patient is receiving long-term care service, *n* (%)	22 (7.9%)	3 (4.3%)

**Table 2:** Characteristics of the patients.

Characteristics	Test Sample (*N* = 280)	Re-Test Sample (*N* = 70)
Age, mean ± SD	60.9 ± 13.2	61.7 ± 14.0
Male, *n* (%)	94 (33.6%)	24 (34.3%)
Married/Cohabiting, *n* (%)	208 (74.3%)	50 (71.4%)
Educational level, *n* (%)		
No formal education	25 (8.9%)	12 (17.15)
Primary education	64 (22.9%)	18 (25.7%)
Secondary education or above	191 (68.2%)	31 (44.3%)
Perceived poor financial status, *n* (%)	86 (30.7%)	25 (27.5%)
Leading types of cancer, *n* (%)		
Breast	79 (28.2%)	16 (22.9%)
Colorectal	52 (18.6%)	19 (27.1%)
Lung	45 (16.1%)	9 (12.9%)
Nose	17 (6.1%)	4 (5.7%)
Prostate	15 (5.4%)	2 (2.9%)
Cervical	14 (5.0%)	3 (4.3%)
Stomach	12 (4.3%)	3 (4.3%)
Others	46 (16.4%)	14 (20.0%)
Year since diagnosis, *n* (%)		
Within 1 year	50 (17.9%)	14 (20.0%)
1–< 2 years	104 (37.1%)	32 (45.7%)
2–< 3 years	49 (17.5%)	11 (15.7%)
≥ 3 years	77 (27.5%)	13 (18.6%)
Currently undergoing treatment, *n* (%)	246 (87.9%)	59 (84.3%)
Type of treatment currently receiving	*N* = 246	*N* = 59
Surgery, *n* (%)	154 (62.6%)	34 (57.6%)
Radiotherapy, *n* (%)	96 (39.0%)	21 (35.6%)
Chemotherapy, *n* (%)	145 (58.9%)	40 (67.8%)
Target therapy, *n* (%)	51 (20.7%)	11 (18.6%)
Hormone therapy, *n* (%)	33 (13.4%)	6 (10.2%)
Others, *n* (%)	8 (3.3%)	3 (5.1%)

**Table 3:** Descriptive statistics, ordinal alpha coefficients, and ICC for each SPUNS-SF domain (*N* = 271).

Domain of SPUNSF	Number of Items	Mean (0–4)	SD	Median (IQR)	Lowest Score (Floor)	Highest Score (Ceiling)	Ordinal Alpha Coefficient	ICC (95% CI)
Information	6	1.26	0.94	1.67 (1.5)	33 (12.2%)	3 (1.1%)	0.912	0.537 (0.256–0.713)
Future Concerns	3	1.87	1.16	2.0 (2.0)	33 (12.2%)	17 (6.3%)	0.945	0.524 (0.230–0.705)
Work and Finances	5	0.96	0.92	0.8 (1.4)	68 (25.1%)	3 (1.1%)	0.873	0.678 (0.477–0.801)
Healthcare Access and Continuity	5	1.77	1.02	1.8 (1.4)	23 (8.5%)	2 (0.7%)	0.866	0.636 (0.412–0.775)
Personal and Emotional Needs	7	1.28	0.85	1.14 (1.14)	21 (7.7%)	1 (0.4%)	0.916	0.598 (0.351–0.751)

ICC, Intra-class correlation coefficient; SPUNS-SF, Support Person’s Unmet Needs Survey—Short Form; IQR, Interquartile range.

**Table 4:** Correlations matrix for construct validity.

	Unmet Supportive Care Needs				
	Information	Future Concerns	Work and Finances	Healthcare Access and Continuity	Personal and Emotional Needs
PSQI	0.189 **	0.121 **	0.273 **	0.075	0.464 **
CSI	0.257 **	0.412 **	0.346 **	0.320 **	0.446 **

PSQI, Pittsburgh Sleep Quality Index; CSI, Caregiver Strain Index; ** *p*-Value < 0.01, * *p*-Value < 0.05.

## Supplementary Materials

The following are available online at https://www.mdpi.com/1660-4601/16/21/4103/s1, Table S1: The Hong Kong Chinese version of SPUNS-SF.
