# Supplementary material for: Validation of the Hong Kong Chinese version of the Support Person’s Unmet Needs Survey—Short Form
Source: Int J Environ Res Public Health. 2019 Oct 24;16(21):4103. doi: 10.3390/ijerph16214103 (PMC6862032; doi:10.3390/ijerph16214103)
Supplement: Supplementary file 1 [file ijerph-16-04103-s001.pdf]

回答指引

我們想了解**在過去一個月內**，你由於幫助曾患上或正患有癌症的人有過的未滿足需求。未滿足需求是指那些需求你一直無法得到滿足。

回答每條問題時，試回想你**過去一個內**未滿足需求的程度，並圈上最能貼切形容的數字。請回答所有問題，即使你認為那個問題是不可能解決到的。

0	**沒有未滿足需求** — 幫助癌症患者並沒有對我造成此問題。
1	**低度未滿足需求** — 我需要小量的協助來解決此問題，但我得不到。
2	**中度未滿足需求** — 我需要中量的協助來解決此問題，但我得不到。
3	**高度未滿足需求** — 我需要大量的協助來解決此問題，但我得不到。
4	**極高度未滿足需求** — 我需要非常大量的協助來解決此問題，但我得不到。

例子

請就下面每一項陳述，圈上最能貼切形容你未滿足需求程度的選項。					
	沒有未滿足需求	低度未滿足需求	中度未滿足需求	高度未滿足需求	極高度未滿足需求
知道如何能坦誠和我幫助的癌症患者談論癌症的話題	0	1	(2)	3	4

如果你圈上 2，表示在**過去的一個月**，你想知道如何能坦誠和你幫助的癌症患者談論癌症的話題，對此有中度需要，但你並未能得到相關的資訊和協助。

圈上最能貼切形容你未滿足需求程度的選項					
尋找和得到經濟援助	(0)	1	2	3	4

如果你圈上 0，表示在**過去的一個月**，對你來說,尋找和得到經濟援助不是問題

我們了解你的未滿足需求可能會隨著時間改變。本調查只旨在了解你過去一個月內你遇到的未能滿足需求。

請前往下頁，並完成問卷。

甲、未滿足的資訊需求: 這部分旨在了解過去一個月內有關尋找資訊的未滿足需求。

請就下面每一項陳述，圈上最能貼切形容你未滿足需求程度的選項。		沒有未滿足需求	低度未滿足需求	中度未滿足需求	高度未滿足需求	極高度未滿足需求
Q1.	尋找有關如何在家中處理這個疾病的資訊	0	1	2	3	4
Q2.	尋找有關癌症和其對性關係影響的資訊	0	1	2	3	4
Q3.	能夠和醫護人員坦誠談論自己的感受和擔憂	0	1	2	3	4
Q4.	尋找有關另類療法的資訊	0	1	2	3	4
Q5.	尋找有關療法和藥物長期副作用的資訊	0	1	2	3	4
Q6.	知道如何能坦誠和我幫助的癌症患者談論癌症的話題	0	1	2	3	4

乙、*未來： 這部分旨在了解過去一個月內有關未來的未滿足需求。*

請就下面每一項陳述，圈上最能貼切形容你未滿足需求程度的選項。		沒有未滿足需求	低度未滿足需求	中度未滿足需求	高度未滿足需求	極高度未滿足需求
Q7.	處理癌症復發的憂慮	0	1	2	3	4
Q8.	處理癌症惡化的憂慮	0	1	2	3	4
Q9.	處理未來不可預知的事情	0	1	2	3	4

丙、未滿足的工作與財務需求：這部分旨在了解過去一個月內有關工作與財務的未滿足需求。						
請就下面每一項陳述，圈上最能貼切形容你未滿足需求程度的選項。		沒有未滿足需求	低度未滿足需求	中度未滿足需求	高度未滿足需求	極高度未滿足需求
Q10.	令我的老闆更支持和了解我	0	1	2	3	4
Q11.	能夠繼續工作	0	1	2	3	4
Q12.	支付非醫療開支(如：交通、特別食物)	0	1	2	3	4
Q13.	處理同事對我現時情況的感受	0	1	2	3	4
Q14.	尋找和得到經濟援助	0	1	2	3	4
丁、對於持續照顧的未滿足需求： 這部分旨在了解過去一個月內有關醫療護理服務的未滿足需求						
請就下面每一項陳述，圈上最能貼切形容你未滿足需求程度的選項。		沒有未滿足需求	低度未滿足需求	中度未滿足需求	高度未滿足需求	極高度未滿足需求
Q15.	接觸到各種的健康護理服務和提供服務的醫護人員（如：營養師、物理治療師、職業治療師）	0	1	2	3	4
Q16.	很快預約到提供服務的醫護人員	0	1	2	3	4
Q17.	確保在每次的跟進中，我幫助的患者能夠看到同一位醫護人員	0	1	2	3	4
Q18.	和醫生有足夠的見面時間	0	1	2	3	4
Q19.	很快取得我幫助的患者的檢查結果	0	1	2	3	4

戊、未滿足的個人和情緒需求：這部分旨在了解過去一個月內與你個人有關的未滿足需求

請就下面每一項陳述，圈上最能貼切形容你未滿足需求程度的選項。		沒有未滿足需求	低度未滿足需求	中度未滿足需求	高度未滿足需求	極高度未滿足需求
Q20.	睡得不好	0	1	2	3	4
Q21.	處理壓力	0	1	2	3	4
Q22.	處理對家庭成員心理健康的憂慮	0	1	2	3	4
Q23.	處理家中雜務（如：煮飯、清潔、家居維修）	0	1	2	3	4
請就下面每一項陳述，圈上最能貼切形容你未滿足需求程度的選項。		沒有未滿足需求	低度未滿足需求	中度未滿足需求	高度未滿足需求	極高度未滿足需求
Q24.	知道我已盡我所能	0	1	2	3	4
Q25.	處理我的感受, 如我令到所幫助的患者失望	0	1	2	3	4
Q26.	尋找這段經歷的意義	0	1	2	3	4
